# Representations of changing weather conditions and outdoor work in the Swedish media: Legitimization of a risk discourse

**DOI:** 10.1371/journal.pone.0315177

**Published:** 2025-02-06

**Authors:** Bo Nilsson, Jenny Lönnroth

**Affiliations:** Department of Culture and Media Studies, Umeå University, Umeå, Sweden; Universidade de Santiago de Compostela, SPAIN

## Abstract

Accelerating climate change has been associated with, among other things, rising temperatures, rising sea levels, extensive periods of precipitation, and difficult wind conditions. These are said to affect large segments of society, not least outdoor workers, whose working conditions are negatively affected. Much media attention has been paid to the situation of outdoor workers, and the media presents tips on what to consider when working in conditions such as high temperatures. The aim of this paper is to explore how the Swedish media reports on outdoor workers and their working conditions in relation to climate change and difficult weather conditions, especially high temperatures. The aim is also to describe and analyze how an identified risk discourse is legitimized in media representations of extreme weather and outdoor work. The study is based on a qualitative analysis of 72 articles in the Swedish media, available in the digital archive retriever.com (Mediearkivet). A social constructionist perspective is used to explore how weather, climate, and risks are “constructed” in the material, and a focus on storytelling techniques and legitimization strategies makes it possible to investigate how certain views of weather, climate, and risks are justified. According to the results, climate change and changing weather conditions are related to risks at both a structural level and an everyday level in working life. Two overall categories of risk are identified, on one side physiological and mental risks, and on the other economic and technological risks. Together, these categories underpin a general risk discourse that is legitimized using strategies such as scientification, dramatization, and personification. The conclusion is that the media representations are characterized by a de-politization of climate change and changing weather conditions, and by a focus on individual adaptation to these changes.

## Introduction

Changed weather conditions, such as rising temperatures and sea levels, increased precipitation, and severe wind conditions are often described, experienced, and understood as signs of a changing climate. These changes are said to affect large parts of society, not least outdoor workers, whose working conditions are negatively affected. The high temperatures in the summer of 2023 resulted in protests by outdoor workers. The Swedish media reported that garbage workers in Rome threatened to strike and that staff at the Acropolis in Athens refused to work during the hottest hours of the day. According to the media material, President Biden commented on the situation for outdoor workers in the following way:

For the farm workers, who must harvest crops in the dead of night to avoid the high temperatures, or farmers who risk losing everything they planted for the year, or the construction workers, who literally risk their lives working all day in blazing heat, and in some places don’t even have the right to take a water break […] That’s outrageous [1].

The Swedish media has also addressed the situation of Swedish outdoor workers in relation to changing weather conditions, and presents tips on what to consider when working in conditions such as high temperatures. This paper is about how outdoor work is described in the Swedish media in relation to climate change, extreme weather, and high temperatures.

## Previous research

This study relates to three fields of research; climate change and working conditions, media representations of climate change, and risks in media representations of climate change.

### Climate change and working conditions

This study is related to investigations of how climate change, especially rising temperatures, affect work and working conditions in different sectors of society [2]. As shown in previous research, globally, many occupational groups are being negatively affected by climate change and rising temperatures, for example in the mining industry [3]. Particularly vulnerable groups include construction and road workers, as well as healthcare and agricultural workers [4–7]. A problem for road and construction workers is that they often work outdoors without protection [8]. According to US studies, construction workers are more exposed to heat-related illnesses than employees in other sectors, and road workers are especially at risk [9, 10]. Changing weather conditions can also mean that professionals are exposed to other hazards; for example, strong winds on building construction sites [11]. Another concern is that Sweden has been described as late in implementing climate adaptation measures in the labor market in general [12]. Climate change and rising temperatures can thus have many different consequences, such as affecting people’s physiological and psychological health [13]. They also have socio-economic consequences, and reduce productivity [14–16].

### Media representations of climate change

This paper is also related to an extensive research field that deals with media representations of extreme weather conditions, climate change, and rising temperatures. The media—both traditional and new media—are often described as key actors in producing and transmitting information across various social domains. “Mediatization” refers to the media as a pervasive societal phenomenon that permeates nearly all facets of modern, global societies, shaping these societies through its unique logic. However, mediatization is not a linear process, and its impact can vary. For instance, individuals who rely on multiple online news sources tend to be less susceptible to the agenda-setting effects of traditional media [17].

However, the influence of the media applies not least to people’s perceptions of climate change [18]. One reason for this is that not everyone is directly affected by climate change but receives information about it mainly via the media [19, 20]. The number of media reports about climate change has also increased over the years [21] and raised people’s awareness—although the impact of the media cannot be described as unambiguous [22], especially because there are examples of the media trivializing the climate issue by avoiding questions of causation and possible impacts.

Characteristic of many media representation of climate change is a dramatizing and alarmist stance with a focus on disasters, scares, and dangers [23]. Journalists tend to make direct connections between dramatic weather events and global climate change [24], which means that they ignore the complexity and uncertainty that pervade scientific knowledge production about climate change [25]. However, this does not mean that all journalists and all media are characterized by scaremongering. In a study of the Argentinian press, Mercado [26] noted that a disaster scenario was reflected in 25 percent of the articles, and almost always in relation to scientific reports and extreme weather events. It has also been shown that some journalists lack the appropriate skills and training to be able to report on climate change based on scientific perspectives and policies [27, 28]. In addition, many journalists are expected to cover several different areas and do not have enough time for more in-depth reporting [29, 30]. As discussed in a study of Swedish journalists [31], this development can be related to growing demands for multi-tasking as well as conflicting tendencies in journalism such as fragmentation versus homogenization and professionalization versus commercialization.

However, the media do not reflect reality in an objective manner but are involved in the social construction of this reality. Here, ideological aspects are significant for how this construction takes place. In a review, Maeseele and Pepermans [32] write about five ideological aspects: economic factors, journalistic norms, political context, ideological cultures, and citizen decoding. The authors argue that all of these factors can be understood as “ideological filters that shape the identities, worldviews, and belief systems that are implicitly or explicitly present in mediated public discourse about climate change” [32]. Furthermore, representations of climate change are influenced by the ideological bias of media companies. Conservative media outlets tend to oppose the concept of climate change and more strongly support climate skepticism [33].

### Risks in media representations of climate change

“Risk” in a broad sense is central to media representations of climate change [34]. A recurring theme in media studies is an examination of the tension between scientific, political, and policy discourses regarding the risks of climate change. This tension can be partly explained by norms of professional journalism and everyday journalistic practices which, according to Boykoff and Boykoff [35], result in an informational bias that “has helped to create space for the US government to defray responsibility and delay action regarding climate change.”

Studies of risk communication have illustrated that the media often amplifies risks, and that the media is a key actor in shaping people’s perceptions of risks [36]. In other words, mediated information is central to people’s awareness of, attitudes towards, and actions in relation to risks [37]—and visual media representations can contribute to the concretization and actualization of risks [38]. From a more theoretical perspective, it can be said that the media are involved in the social construction of risks; that is, they are involved in creating, recreating, and mediating risks [23]. However, social media, in particular, plays a key role in challenging mainstream perspectives on climate change and its associated risks, and arguments from climate skeptics are common on various websites and blogs [39].

Risk communication about extreme weather and climate change (EWCC) is a challenging practice because people relate in different ways to this type of communication. As shown by Pilla et al. [40], it is difficult to reach out with information about the risk of flooding, because people are more focused on previous floods than scientific assessments of the risk of new ones. Under extreme weather conditions, argue Blashki et al. [41], risk communication tends to deal with short-term measures, while climate change requires a long-term proactive risk communication that focuses on aspects such as the adaptation of society’s infrastructure.

The framing of extreme weather and climate change is important for the effect of risk communication. As illustrated in previous research, if climate change is perceived as imminent, it can lead to increased anxiety [42], as well as to people becoming more inclined to change their behavior [43], for example by reducing their energy consumption [44, 45]. The affective and emotional component of a message has also been considered central to the effectiveness of risk communication [46].

Wachinger et al. [47], who reviewed the literature on natural risks, noted that personal experiences of events such as floods, droughts, or forest fires, together with an established trust in communications from the authorities and experts, had the greatest impact on people’s risk perceptions. People’s risk perceptions regarding climate change are also dependent upon categories such as age, education, and gender [48].

An “advantage” of the risk discourse is that it can confirm the idea of climate change as an urgent problem—it is no longer possible to postpone measures. “Risk” can also enable a more nuanced approach compared to words like “disaster” [49].

Thus, there are several studies about climate change, media representations of climate change, and the risks associated with such change. However, there are very few studies focusing on media representations of concrete risks for outdoor workers in relation to changed weather conditions and rising temperatures. As stated by Moda, Filhos and Minha [2], more research is needed about how climate change affects outdoor work, which this explorative study aims to provide. The paper is also an important contribution to research on how outdoor work is (re)constructed in the media at the intersection of climate change, risk perception, and risk management.

## Aim

The primary aim is to explore how the Swedish media reports on outdoor workers and their working conditions in relation to difficult weather conditions—especially high temperatures—and climate change. The secondary aim is to describe and analyze how an identified risk discourse is legitimized in media representations of extreme weather and outdoor work, and to discuss possible consequences of these representations and the legitimized risk discourse.

## Materials and methods

This paper is based on media material available in the digital database retriever.com (Mediearkivet), which means that it only includes published data. The database retriever.com enables searches in 4071 different Swedish media outlets from 1945 onwards (print media, online media, radio and TV, and podcast). We made searches using the keywords “klimatförändring AND utomhusarbete (“climate change” AND “outdoor work”), “storm AND utomhusarbete” (“storm” AND “outdoor work”), “nederbörd AND utomhusarbete” (“precipitation” AND “outdoor work”), “klimat AND utomhusarbete” (“climate” AND “outdoor work”), “kyla AND utomhusarbete” (“cold” AND “outdoor work”), and “värme AND utomhusarbete” (“heat” AND “outdoor work”). These resulted in 2, 7, 11, 72, 118, and 125 hits respectively (total 335). The searches targeted all types of media without a defined time period, and the results include newspapers, online newspapers, online magazines (e.g., health magazines and working-life magazines), as well as online articles published by public service radio (Sveriges Radio) and public service television (SVT). The variety of media types allows them to reach diverse target groups. The first hits were from the mid-1990s and continued until the present. Most of the hits were from the 2010s onwards. We made a strategic selection from among the 335 hits, based on the guiding principle that we wanted data that encompassed argumentative statements about weather and outdoor work, and did not just briefly mention these phenomena. This strategic selection resulted in 72 hits and thus meant that we excluded several of the texts, including duplicates, found in the media material.

It is characteristic of the material in general that it deals with changed weather conditions and rising temperatures in relation to outdoor work. Climate change is mentioned, but not to the extent that we had expected. However, it seems that the writers were taking climate change as an implicit guiding star in their writing.

The material has undergone a qualitative analysis, characterized by an interest in processes of meaning making, the interpretation of symbolic material, and (the possibility of) different interpretations [50]. An important analytical point of departure has been to consider the media material as representations. This means that the articles, which are text-based and give the impression of representing an objective reality, have been treated as governed by conventions, genres, and stylistic expressions [51].

The concrete analysis was divided in two stages. In stage one we were inspired by Braun and Clarke’s [52] analytic process consisting of six phases. In the first phase, we familiarized ourselves with the data, which meant that we repeatedly read the articles in order to gain an in-depth understanding of each text. We also assessed the quality of the data, whether the articles were ambiguous or contradictory. In the second phase, initial codes were identified by focusing on recurring notions and concepts that conveyed similar meanings, and that often related to problems with climate change and difficult weather conditions. The coding phase resulted in categories such as “health problems”, “reduced productivity” and “deficient technologies”. In phases 3–5 we searched for themes, reviewed themes, and defined and named themes based on similarities between the identified categories. A common similarity was notions of danger and threat, which we perceived as expressions of “risk” as the main theme. “Physiological and mental risks”, “technological and economic risks” and “an increased crisis” were defined as sub themes. Braun and Clarke’s sixth phase, producing the report, was completed after stage 2 in the analytical process.

During the first stage of the analysis, we noticed that the authors of the media texts used rhetorical techniques when arguing for different risks of climate change and difficult weather conditions. Therefore, in stage two of the analysis, we focused on identifying storytelling techniques such as simplification, polarization, intensification, concretization, personification, and stereotyping [53, 54]. These techniques often have the purpose or effect of reducing the amount of information—there is an excess of media information in general—and capturing people’s attention [55]. However, these same techniques can also be used to legitimize or justify different ideas about weather, climate, and temperatures; for example, the writers express an implicit or explicit ambition to convince their readers that rising temperatures and climate change are real and proven phenomena. We came to interpret this ambition in terms of strategies of legitimation: different rhetorical strategies being used with the purpose of justifying or legitimizing a message. Here we were inspired by Reyes [56] and the legitimation strategies that he identified in political rhetoric, such as references to experts and to a hypothetical future. It may be noted that there is a difference between persuasion and legitimation. While the former is usually linked to individual abilities [57], such as the capacity to influence other people, the latter focuses on how credibility is created in a text without involving individuals.

A qualitative analysis can be perceived as “purely” inductive, but in our case, it is probably more accurate to call it abductive, since there has been an interaction between material, theory, and previous research [58]. For example, “risk” is a discourse identified in the material, but it would be misleading to describe this discourse as the result of a strictly inductive process. Both before and during our investigation, we came across texts that deal with the connection between changing weather and (experiences of) risks, and these have influenced our analysis, implicitly or explicitly. The set of concrete examples of what characterizes risks were, however, inductively identified in the material.

The results of the different stages of the analysis were repeatedly compared with the media material and with earlier interpretations in the analytical process to avoid misinterpretations. Furthermore, the two authors discussed the analytical process repeatedly until a consensus was reached to ensure the quality of the analysis [59].

## Theoretical perspectives

In this paper, we adopt a social constructionist perspective, which means that we assume the understanding or interpretation of objects and societal phenomena is dependent upon culture, environment, context, etc., and not (only) on the object and the phenomenon itself [60]. In line with this, we regard climate and weather as socially and culturally constructed phenomena, whereby climate can be described as an idea that humans use to organize the relations between weather and their lives [61]. However, the fact that we regard climate and weather as culturally constructed does not mean that we deny the existence of facts showing, for example, a changing climate over time with rising temperatures, but, according to our theoretical point of view, such facts, and how they are interpreted, used, and communicated, are dependent upon actual (and dominant) ideas about climate and weather:

That we can reduce the complex phenomenon of climate change to a few words and use a rhetorical flourish to give it enormous emotional power tells us that this phenomenon is a social construction and not just a collection of facts about temperatures, emissions, rainfall patterns and the like. Even the facts are for the most part social [62].

For example, talking about weather in terms of rising temperatures and devastating droughts will convey different ideas about climate change than talking about weather and rising temperatures in relation to an extended growing season and increased harvests. Thus, there is a relative component to stories about changed weather conditions and climate change.

Inspired by the social constructionist perspective, we are interested in how risks are “shaped” and presented in the media material. We thus consider risk to be a construction, which means that risks are created in interaction between different actors, ideas, and forces, and should not be seen as something given or fixed, as is often assumed [63]. Risks can be seen as a kind of shared and historically changing agreement, and an agreement that is related to late modern society. As stated by Beck [64], risks as a central organizing principle have substantiated a culture of fear and contributed to the rise of risk-handling companies and organizations, for example insurance and security companies. Media communication about risks usually also has certain purposes, such as informing the public, warning of a threatening future, starting political debates, or obtaining many “clicks” on social media according to the logic of capitalism.

The media logic is important here. As stated by Altheide and Snow [65], journalism is not based on objective investigations of what is happening in “the real world” and what is essential there. Instead, journalism is shaped by the media’s own production conditions, its own media formats, and time rhythms. For example, according to the media logic, it is important to avoid (too much) complexity in reports. Moreover, the social reality that journalists write about is already adapted to the media logic. There is thus no independent reality that can be investigated. Altheide and Snow’s [65] reasoning can also be seen as an example of how news is socially and culturally constructed.

## Findings: Dangerous weather

Global heating—a threat against working life. (Our translation) [66]

The media material can be divided into two overall genres. The first deals with extreme weather, rising temperatures, and climate change at a structural level; the second addresses the effects of extreme weather conditions at a concrete, lived level, in everyday working life. Common to the media representations, however, is the fact that extreme weather and high temperatures are perceived as crucial and global problems for humanity. In the long term, everyone is said to be affected by climate change and high temperatures, even though outdoor workers and so-called risk groups are described as the most vulnerable populations. Information stating that more than half of the world’s workforce works outdoors, in the agricultural sector, and in construction and manufacturing industries [67], illustrates that many people are dependent upon the weather. Such information also has the effect of reproducing perceptions of risk in terms of danger and threat, and in the following we address different overall categories (themes) of risk in the media material: physiological and mental risks, and economic and technological risks. These themes can also be supplemented with a temporal dimension, in the sense that the risks are expected to increase over time.

### Physiological and mental risks

Work in intense heat is stressful for the body and can be downright dangerous. This means, among other things, extra work for the heart. In combination with heavy work and/or high humidity, high temperatures can be harmful. (Our translation) [68]

This quote illustrates that high temperatures in connection with (physical) work pose a risk, or a threat, to human health. More concretely, it is claimed that high temperatures can increase the risk of dehydration, exhaustion, heat stress, skin redness, headache, nausea, muscle pain (due to significant salt losses), pain in the back and joints, impaired blood circulation, etc.: “In the extreme, you can suffer from heat stroke, which can be directly life-threatening” (Our translation) [69]. Sun and heat are also linked to skin cancer: “Every year more than 25,000 Swedes get skin cancer caused by the sun. And the cases are increasing” (Our translation) [70].

According to the media material, so-called risk groups are particularly vulnerable; for example, people with cardiovascular disease, kidney problems, severe obesity, multiple sclerosis (MS), and diabetes. Certain medicines and alcohol are said to increase the risks, and factors such as “…age, gender, pregnancy, infections, ability to acclimatize, physical fitness, body size and body composition” (Our translation) [71] are linked to a heightened sensitivity to high temperatures.

It is further stated that absence from work due to illness increases in high temperatures, at least according to an article dealing with the situation in Holland:

Sick this summer? It could be due to the heatwave. In Holland, absence due to illness is extremely high right now, and the reason is said to be physical problems caused by the heat. (Our translation) [69]

High temperatures are also associated with mental risks. Heat is said to affect attention, judgment, and reaction skills, and can cause workers to feel light-headed, which increases the risk of injury and accidents. Furthermore, it is emphasized that people’s mood can be affected by heat, which can cause increased irritability and worsen the psychosocial work environment in general. An interviewed barista says:

…I get a headache. I also notice that I become a little shorter in my tone towards others. It’s not good if you’re a person like me who works in a service profession, she says. (Our translation) [72]

A recurring theme in the media material is thus risk production, whereby rising temperatures and outdoor work are linked to various threats to human health. The result is that heat and outdoor work are primarily constructed as a dangerous and hazardous combination.

### Economic and technological risks

Financial and technological risks are usually treated at a general level in the media representations. It is stated, for example, that both individual nations and the whole world could suffer financially due to a changed weather situation. Economic risks include reduced productivity and lower GDP, and these are commonly mentioned in reports from countries that suffer even more from high temperatures than Sweden does. One example is India, and it has been stated that the country has suffered from an increasing number of heat-waves, which have negatively affected both outdoor workers and productivity:

By 2030, heatwaves could cost India up to 4.5 percent of the country’s GDP in the form of reduced productivity […] By 2050, some parts of India are expected to be exposed to such heat and humidity that outdoor work is practically impossible during almost 30 percent of the year’s working hours during daytime [….] the number of daily working hours that will be lost due to reduced productivity will rise by around 15 percent by 2030. (Our translation) [73]

Here it is stated that “outdoor work is practically impossible”. Similarly, the work environment for field technicians in Sweden in 2018 was described as marked by “unbearable heat” [74], which was also considered to have concrete practical consequences: the work became much harder and took longer to complete. In other words, the heat resulted in reduced productivity at the same time as the field technicians suffered from “unbearable heat”.

Rising temperatures are also considered to be important for technologies, both today and in the future. For example, trains are reported to be negatively affected by heatwaves, due to an increased number of so-called solar curves and because the heat challenges the technical quality of the vehicles. But also, rail workers are affected and subjected to increasingly long workdays in the heat, and train attendants (just as passengers) suffer from inadequate air conditioning. Consequently, outdated and insufficient technologies pose risks for staff, while the need for new technologies introduces financial risks due to the significant investments required.

### A temporal dimension: An increasing crisis

The risks have a temporal or dystopian dimension in that they are expected to increase over time unless global warming is stopped:

According to the researchers, more summers with prolonged extreme heat and drought, with forest fires as a result, are to be expected. But also, more precipitation during the winter months with rain in southern Sweden and heavy wet snow in the north. It will have consequences on all levels, not least in professional life. (Our translation) [74]

This article also predicts how different occupational groups will be affected. It is stated, for example, that, with rising temperatures, it may become necessary for road workers to escape the worst of the heat by working during the night. However, such a development is also linked to negative consequences for the work environment.

Contributing to the perception of risk are reports stating that the labor market and many industries are unprepared for the consequences of heatwaves, and that there are no routines in place to help people cope with high temperatures: “Every time there is a heat wave, many people seem to be surprised. They are not prepared for it” (Our translation) [12]. Similarly, it is emphasized that: “Heat waves can appear relatively unexpectedly. Then you do not have time to acclimatize or change routines quickly enough” (Our translation) [75].

### Strategies of legitimation

As stated above, journalists legitimize the notions of changing weather conditions in terms of dangers and threats by specifying various risks and emphasizing their extent. In the following, we describe specific legitimation strategies that reproduce notions of changing weather and high temperatures as risks, and motivate a range of risk-management strategies. As stated in the introduction, strategies of legitimation represent rhetorical strategies aimed at justifying or legitimizing an idea or statement.

#### With the support of experts: Scientification

It is characteristic of media representations that journalists present arguments, facts, and figures that make (the changed) weather conditions appear urgent, increasingly common, and an imminent risk. In their arguments, these journalists are often supported by scientific studies, established organizations, and experts. Just the fact that experts and expert organizations are involved in the arguments relating to rising temperatures contributes to risk production. The weather question is thus made into a serious problem, because it needs to be handled by experts.

For example, in the material there are references to Amnesty International and the McKinsey Global Institute (MGI). In a review of a report by the latter, significant economic consequences and reduced productivity are highlighted as being the result of heatwaves [73]. Heat is thus associated with imminent financial risks in a similar way as mentioned above. The author also highlights the World Health Organization (WHO), which estimates that the number of people who die each year due to heatwaves will exceed a quarter of a million by around 2050 if global warming cannot be slowed down. A major risk associated with rising temperatures is thus a significant increase in related deaths.

In addition to referring to established organizations, journalists mention and quote individual and named experts, which is also a way of legitimizing a risk message. In an article, a representative of the US-based Natural Resources Defense Council talks about vulnerable occupational groups and all the different symptoms they exhibit due to high temperatures, such as fainting and blackouts [73]. The enumeration of a multitude of symptoms makes high temperatures into a widespread problem.

Journalists’ tendency to avoid reporting conflicting perspectives can be understood considering the prevailing consensus in Swedish mainstream media, which primarily views climate change as human-caused and scientifically proven [76]. One possible consequence of this approach is a simplification of the climate issue, making climate problems appear more urgent. This can also be seen as a legitimization strategy, where only some ideas about climate change and shifting weather patterns are presented as credible and reliable.

This strategy can be compared to Reyes’ [56] “voices of expertise”, which refers to arguments, beliefs, or organizations being made legitimate with the help of references to experts. There are also parallels to Van Leuwen’s [77] concept of “authorization”, and especially “expert authority”, according to which claims of legitimacy are made with the help of statements by experts.

This is also characteristic of the media material. The sharpening of arguments that takes place with the help of reference to experts is also supported by the fact that journalists usually avoid any critical examination of the experts to whom they refer. Individual statements and reports are regarded as pure facts, and they reproduce a climate discourse according to which weather changes are seen as an urgent problem and an imminent risk.

#### Deadly weather: Dramatization

A related storytelling technique to the above is dramatization, according to which the changing weather conditions are described as something upsetting and almost shocking. A difference from scientification is that dramatization has a more emotional character, which also makes it effective as a legitimation strategy. In the following examples, the danger of heatwaves is emphasized:

Already today, an estimated 12,000 people worldwide die annually in connection with heatwaves. But the World Health Organization, WHO, states that this figure could reach over a quarter of a million dead each year by 2050, if global warming is not stopped. (Our translation) [73]

In this quote, dramatic figures are presented, of around 12,000 deaths per year due to high temperatures. The message is reinforced by the dystopian prediction of a quarter of a million dead by the middle of the century. The media material also comprises reports about several workers in Spain who died while working in temperatures above 40 degrees, and of unbearable conditions for construction workers in Texas, United States. They are said to be suffering from heatstroke and impaired health in general.

A dramatic prediction is that certain occupations will totally disappear from places where it will be too hot: “If the temperature continues to rise at the same rate, it will not be possible to work in certain places in the future. In some parts of the world, outdoor occupations could become too hot for a human presence”. (Our translation) [66]. Extreme heatwaves and record highs above 45 degrees are being reported from Texas. In the same article, it is mentioned that it is legal in the United States to force people to work during extreme heatwaves, and that “during a serious heat wave, [Texas] passed a law to eliminate mandatory water breaks for outdoor work”. (Our translation) [78]. Such information makes the content almost shocking, at least to Swedish readers.

In the media material, events and conditions are dramatized through the journalists’ choice of concepts, for example “deadly heat”, “extreme heat”, and “record high temperatures”. Such concepts make the issue of changing weather conditions appear urgent, and immediate measures thus appear necessary and legitimate. The heat is not only stressful, but also deadly. Dramatization can thus be understood as a legitimation strategy. It is not merely a news technique—like simplification and intensification [53, 54]—intended to capture the reader’s attention. It also legitimizes information about hazardous weather conditions and, by extension, threatening climate change as a set of real and increasingly frightening phenomena by exclusively emphasizing the danger of rising temperatures and by using concepts such as “deadly heat”.

The media representations can also serve as a kind of warning example; that is, when they link ideas about a changed climate to rising temperatures globally, they also constitute a foreshadowing of the ever-increasing risks that could await Sweden in the future. Notions of future and threatening risks also fit with a market-driven journalism, which benefits from presenting dramatic and sensational information.

To provide context, Hallin and Mancini’s (2004) analytical model can be useful. It distinguishes between three media models: Polarized Pluralist, Democratic Corporatist, and Liberal. Sweden and the Nordic countries exemplify the Democratic Corporatist model, characterized by a strong party press, high degree of professionalism, and significant state intervention [79]. However, as Nord (2008) points out, Nordic media systems have gradually shifted toward a more liberal model, with a reduction in party press affiliations and some government press subsidies [80]. Additionally, structural changes have affected Nordic media, as new competitors increasingly challenge major public service companies. As a result, Swedish journalists view news production as becoming more commercialized, with commercial actors gaining influence [81, 82], which may encourage trends toward entertainment and sensationalism at the expense of explanation and in-depth analysis. The dramatization of climate change observed in the media material for this study is thus an example of such trends.

#### Individual testimonials: Personification

The risks are reinforced by the fact that the reports not only deal with climate change and severe weather conditions at a general level, but also describe their effect on individuals. Personification as a strategy of legitimation refers precisely to the tendency to focus on individuals as individuals, and not as representatives of an organization, a company, or an authority [55].

Recurring throughout the media material are reports about and interviews with people who work outdoors and who testify about the problems brought by extreme weather. A sheet metal worker states that the temperature on the roofs where he works can reach up to 60–70 degrees C, which means that the work cannot be carried out to the same quality as at lower temperatures, and that he becomes extremely light-headed: “you can’t concentrate”. (Our translation) [83]. He also says that he drinks five liters of water every day and uses a lot of water substitutes.

Individual testimonies make the dangers of high temperatures appear less abstract and more relatable [42]. The heat affects not only people close to the equator, but also “ordinary” workers in Sweden. Personification can thus be understood as a legitimation strategy because it makes certain knowledge about high temperatures seem valid; that is, it provides knowledge that links heat to danger and threat.

Personification also comprises an opposing tendency in relation to the section on dramatization above, i.e., de-dramatization. De-dramatization is not as pervasive as dramatization, but it does appear on a few isolated occasions when individuals testify that the heat is not a major problem at all. For example, one interviewee says during a heatwave that she prefers the heat to the cold. One of her colleagues agrees: “I like heat more than cold. For me, summer is the most beautiful”. (Our translation) [83].

### Strategies for risk management

A recurring theme in the media material is descriptions of different actions that people can take to protect themselves and others against adverse weather conditions such as high temperatures. We understand such actions in terms of risk management strategies. Inherent in such strategies is the idea that it is possible to avoid undesirable outcomes or that, if they are unavoidable, they can at least be mitigated [84, 85]. The latter is usually the case in the media material, where the strategies range from structural efforts and technological investments in technological solutions to individual micro-measures. An example of the latter is a recommendation in the media material in relation to high temperatures to work in the shade and to schedule strenuous work tasks for the early morning. Other suggested measures are to take more frequent breaks, wear a hat and sun protection, drink cold drinks, and cool the wrists with cold water.

A recurring idea in the media material is that new and more energy-efficient technologies must be developed to aid in risk management. The material is also characterized by a belief in the possibilities of digital technology. An example is an article introducing an app that can protect people against both heat stress and cold stress. The app is connected to digital data on local weather conditions, and by entering personal data such as weight and age, the app can indicate, for example, how much a person needs to drink in high temperatures [67].

The suggested measures for risk management are defensive in the sense that they are not aiming to deal with the source of the problems—i.e., the cause of the temperature changes—but to adapt to these changes. At the same time, the mediated information—stating that heat must be handled in different ways—contributes to making heat appear (even more) fraught with risks. Thus, the proposals for countermeasures involve risk production in themselves.

As will be elaborated on in the discussion, the management strategies are characterized by two themes related to risks: an individual approach to handling adverse weather conditions, and a reliance on technological development.

## Discussion

The media material we examined was characterized by risk production, and different categories of risk due to rising temperatures were identified: physical and mental, as well as economic and technological risks. The idea that climate, weather, and temperatures are changing at a global scale was something that reporters took for granted, and they legitimized the notion of such a changed scenario by referring to experts as well as by dramatization and personification.

Repeated references were made to scientific reports and experts, and journalists accepted these reports and expert opinions “straight up”—they did not question them, or engage in critical investigative journalism. This substantiated the idea of changing weather conditions and rising temperatures as primarily a threat. However, there were a few hints of other perspectives; for example, that changing weather conditions could promote shipping, as less icebreaking is needed, and with rising sea levels the risk of running aground decreases [74]. But such examples of positive effects in some areas were exceptions.

Dramatization as a strategy of legitimation appeared in media representations that predicted a very large increase in the number of climate-related deaths, and emphasized that many places would become uninhabitable due to sharply rising temperatures. Personification as a strategy of legitimation could be identified in articles where individuals described their own experiences of difficult weather conditions, especially high temperatures. Here, too, notions of risk and danger were reproduced, as the heat was believed to cause anything from direct burns to dizziness and nausea. However, there were also a few hints of alternative experiences, when individuals emphasized that high temperatures were appreciated.

It can thus be concluded that the studied media representations are involved in the social construction of risks, which means that, first and foremost, they reproduce and convey ideas about dangers and threats [24]. This construction results in what can be described as a relatively fragmented risk discourse, as the articles differ in terms of content. They cover everything from rising temperatures as a global phenomenon to tips on drinking a lot of water and drinking often when working outdoors in high temperatures. However, the articles are organized by the same nodal point in the risk discourse: the changing weather conditions are exclusively associated with danger and threat.

In the following, we seek to discuss potential consequences of the media representations and the identified risk discourse. An “advantage” of the risk discourse is that it indicates a serious and global threat posed by rising temperatures, and also highlights the importance of acting. It can also, as Painter [49] points out, be more nuanced to speak of “risk” instead of, for example, “disaster,” because the latter can give the impression that the situation is totally hopeless.

Any negative consequences of the media representations are related to the fact that they are characterized by risk communication, and they legitimize ideas according to which climate, weather, and temperatures are changing irreversibly, and exclusively represent dangerous and threatening phenomena. The effect of this can be understood as a depoliticization of these changes, because they are (indirectly) presented as inevitable, and because of the absence of articles demanding that state authorities take measures.

Another closely related consequence of the media representations and the identified legitimation strategies, not least dramatization, is that they reinforce the apocalyptic and global aspects of changing weather conditions—as Allan et al. [36] have stated, they reinforce (the experience of) imminent risk. The affective and emotional nature of these dramatizations, with a focus on intimidation, danger [23], and shocking details about what is to come, also contributes to perceptions of the risks as becoming more and more threatening [46]. The media logic, according to which communication should be clear and avoid the complexity that characterizes, for example, scientific communication, equally contributes to perceptions of the risks as increasingly serious.

The abundance of similar information about (the effect of) changing weather conditions can work against the explicit or implicit intentions of the media representations, which are to counteract climate change. As stated by Moser and Dilling [86], the strategy of disseminating more information about climate change has no significant effect on people in general. The same applies to information that is based on expert opinions and creates fear. Such information has no significant effect in terms of generating acceptance of the climate threat or increasing people’s support for climate adaptation measures. Instead, the media’s approach to risk communication can have a negative impact on people and their experiences of being able to do anything at all about climate change. As discussed in psychological studies, uncertain prospects mean that people distance themselves from climate change as a real and threatening phenomenon, which also reduces their propensity to engage in climate adaptation measures [87, 88]. At the same time, one cannot totally ignore Roeser’s [89] ideas that emotions are central to effective risk communication, because they can contribute to a deeper understanding of the moral aspects of climate change and contribute to sympathy with its victims. Emotions can also function as a more effective motivator than rational and abstract knowledge [46].

According to Blashki et al. [41], risk communication in relation to extreme weather is usually about short-term measures, but what is really needed is long-term proactive risk communication that focuses on climate adaptation of society’s infrastructure in the long run. The short-term perspective is reflected in the analyzed media material through the advocated risk-management strategies, which focused on how outdoor workers in everyday life can master and alleviate the stress of high temperatures. Suggested measures included everything from varying work tasks to taking long breaks and drinking water regularly. These measures are, of course, understandable from a work environment perspective, but they also focus on short-term adaptation to climate change, not on measures to slow it down. Thus, as mentioned above, the suggested measures give the impression of climate change as an irreversible phenomenon, and that the only thing to do seems to be to accept it and try to handle the changes.

The presentations of tips and advice were also characterized by a high degree of concreteness, such as the suggestion that outdoor work should take place in the shade. Advantages of this concretization are, of course, that the risk of health problems may be reduced, but the enumeration of many different concrete countermeasures can in themselves result in heightened risk perception, just because there are so many. Another aspect of these risk management strategies is that some of them individualize the problems of changing weather conditions and rising temperatures [90]. It is, they seem to imply, primarily up to the individual to take steps to prevent harm.

In previous research, it has been established that there is a strong belief across society that new technologies and technical innovations will solve the climate crisis. For example, Hornborg [91] writes about technology fetishists, i.e., about the strong belief in (new) technology, for example among people in power, as the ultimate climate crisis solution. However, Hornborg also emphasizes that this belief turns a blind eye to the fact that even new technologies result in stress on the climate [91].

In the media material technology is certainly highlighted. But rather than as the solution to climate crises, technological development is described as a necessity in order to adapt to new weather conditions. However, any negative aspects of technological development are not discussed. According to these media representations, it seems as though people can almost continue to live their lives as before based on a few technological adjustments—besides the abovementioned everyday adaptation strategies. Technological development in general seems to be organized by a “logic of efficiency,” according to which it is claimed that people will be able to increase production and consumption without negatively affecting the climate and nature. However, as stated by, for example, Hornborg [91], new and so-called green technologies are not the solution to the problems of climate change, because many of these technologies are based on fossil fuels, and large amounts of metal and plastics.

One conclusion is that Swedish media representations of outdoor work, changing weather conditions, and climate change reproduce the risk discourse identified in previous research. What this study adds, however, is an in-depth account of how this discourse is legitimized and of its “unintended” effects.
